# Gut Microbiota during Dietary Restrictions: New Insights in Non-Communicable Diseases

**DOI:** 10.3390/microorganisms8081140

**Published:** 2020-07-28

**Authors:** Emanuele Rinninella, Marco Cintoni, Pauline Raoul, Gianluca Ianiro, Lucrezia Laterza, Loris Riccardo Lopetuso, Francesca Romana Ponziani, Antonio Gasbarrini, Maria Cristina Mele

**Affiliations:** 1 UOC di Nutrizione Clinica, Dipartimento di Scienze mediche e chirurgiche, Fondazione Policlinico Universitario A. Gemelli IRCCS, Largo A. Gemelli 8, 00168 Rome, Italy; 2 Scuola di Specializzazione in Scienza dell’Alimentazione, Università di Roma Tor Vergata, Via Montpellier 1, 00133 Rome, Italy; marco.cintoni@gmail.com; 3 UOSD di Nutrizione Avanzata in Oncologia, Dipartimento di Scienze mediche e chirurgiche, Fondazione Policlinico Universitario A. Gemelli IRCCS, Largo A. Gemelli 8, 00168 Rome, Italy; pauline.raoul1@gmail.com (P.R.); mariacristina.mele@unicatt.it (M.C.M.); 4 UOC di Medicina Interna e Gastroenterologia, Dipartimento di Scienze mediche e chirurgiche, Fondazione Policlinico Universitario A. Gemelli IRCCS, Largo A. Gemelli 8, 00168 Rome, Italy; gianluca.ianiro@hotmail.it (G.I.); laterza.lucrezia@gmail.com (L.L.); lopetusoloris@libero.it (L.R.L.); francesca.ponziani@gmail.com (F.R.P.); antonio.gasbarrini@unicatt.it (A.G.); 5 Department of Medicine and Ageing Sciences, Faculty of Medicine and Surgery,“G. d’Annunzio” University of Chieti-Pescara, Via dei Vestini 31, 66100 Chieti, Italy; 6 Center for Advanced Studies and Technology (CAST), “G. d’Annunzio” University of Chieti-Pescara, Via dei Vestini 31, 66100 Chieti, Italy; 7 Dipartimento di Medicina e Chirurgia traslazionale, Facoltà di Medicina e Chirurgia, Università Cattolica Del Sacro Cuore, Largo F. Vito 1, 00168 Rome, Italy

**Keywords:** gut microbiota, caloric restriction, intermittent fasting, fasting-mimicking diet, non-communicable diseases, aging

## Abstract

In recent decades, there has been a growing interest in dietary restrictions for their promising effects on longevity and health span. Indeed, these strategies are supposed to delay the onset and burden of non-communicable diseases (NCDs) such as obesity, diabetes, cancer and neurological and gastrointestinal inflammatory diseases. At the same time, the gut microbiota has been shown to play a crucial role in NCDs since it is actively involved in maintaining gut homeostasis through its impact on nutrients metabolism, gut barrier, and immune system. There is evidence that dietary restrictions could slow down age-related changes in the types and numbers of gut bacteria, which may counteract gut dysbiosis. The beneficial effects on gut microbiota may positively influence host metabolism, gut barrier permeability, and brain functions, and subsequently, postpone the onset of NCDs prolonging the health span. These new insights could lead to the development of novel strategies for modulating gut microbiota with the end goal of treating/preventing NCDs. This review provides an overview of animal and human studies focusing on gut microbiota variations during different types of dietary restriction, in order to highlight the close relationship between gut microbiota balance and the host’s health benefits induced by these nutritional regimens.

## 1. Introduction

For centuries, fasting has been proposed by almost all religions of the world for its beneficial effects on the spiritual life [1]. The modern industrialized society has lost this habit, promoting the continuous and excessive intake of food, often junk food, as a form of bodily wellbeing and self-gratification. However, unhealthy food, excessive caloric intake, and inactivity are clearly related to non-communicable diseases (NCDs) such as obesity, diabetes mellitus, cancer, and cardiovascular and other inflammatory conditions [2]. The prevalence of NCDs has increased over recent decades with 41 million deaths each year, equivalent to 71% of all deaths globally [3].

In this context, diet plays a part in the management of NCDs. Indeed, Mediterranean diet can reduce the incidence of cardiovascular diseases, cancers, neurodegenerative diseases, and diabetes [4]. In addition, dietary restrictions in meal frequency and/or portion size could potentially represent effective therapies in preventing and delaying the onset of NCDs [5,6,7]. To date, several approaches to dietary restriction have been evaluated. First, caloric restriction (CR), defined by a 20–50% reduction in energy without occurring malnutrition or reduction in essential nutrients, was studied in several animal and human experimental trials such as the multi-center Comprehensive Assessment of Long-term Effects of Reducing Intake of Energy (CALERIE) [8]. CR could represent a safe and feasible intervention to improve and prevent NCDs during human aging. However, several limitations were suggested in terms of long-term compliance [9], inter-individual variability in body mass [10], and safety in case of surgery and injury [11]. Recently, the control of feeding and fasting time has become an alternative of great interest. The most studied schedules are (i) time-restricted feeding (TRF) which provides food intake in a 4- to 12-h time window, (ii) intermittent fasting (IF) which provides an alternation of 24-h fasting (or very low calories, corresponding to 25% of energy needs) with a 24-h ad libitum eating period, and (iii) fasting-mimicking diet (FMD) proposing a reduction of caloric intake for five consecutive days, through a low-caloric vegetable-based diet, before returning to normal eating cycles once a month. Many studies suggest promising beneficial health effects by performing these strategies, such as the delay of the onset of chronic diseases in selected patients [12,13,14,15].

In parallel, numerous studies have reported changes in microbiota composition in both preclinical and clinical models during dietary restrictions [16,17,18,19,20,21,22,23,24]. Gut microbiota is a complex community of over 100 trillion bacteria, yeasts, viruses, and parasites influencing host physiology, metabolism, and immune function [25]. Besides its well-known beneficial functions, the gut microbiota can also turn detrimental to its host and, if not well-managed, lead to the onset of metabolic and inflammatory disorders. Indeed, gut microbiota dysbiosis is associated with several intestinal diseases, such as inflammatory bowel disease (IBD) [26] and colorectal cancer [27], as well as extra-intestinal diseases, such as neurological disorders [28].

This review aims to dissect the impact of different dietary restriction strategies on gut microbiota homeostasis and highlight the consequences on host health. Furthermore, we will describe the gut microbiota variations induced by these dietary restricted regimens. Finally, we will see how gut microbiota may play a role in the crosstalk between dietary restrictions, health benefits, and NCDs.

## 2. Dietary Restrictions in Health and NCDs

### 2.1. Cellular Responses to Dietary Restrictions Implicated in Longevity Pathways

To date, much evidence has indicated the positive effects of dietary restriction on the lifespan of multiple species [6,10,29,30,31]. Recently, the analysis of single cells in rats during CR showed that CR attenuated aging-related changes in cell-type composition, gene expression, and core transcriptional regulatory networks [32].

Fasting time and energy restriction share biological cellular defense responses resulting in lifespan extension [33,34,35]. Reduction of calories by energy restriction or fasting periods triggers an increase of circulating ketones whereas amino acids, glucose, and insulin are maintained at low concentrations [9]. In rodents, CR may induce an increase of growth hormone (GH) levels and decreased insulin-like growth factor (IGF-1) levels [36]. Downregulation of IGF-1 signaling pathway and the reduction of amino acids circulation could repress the activity of the mammalian target of rapamycin (mTOR), leading to an overexpression of sirtuin-1 (SIRT-1) [9,35]. mTOR is a key component of cellular metabolism regulating several hallmarks of aging such as energy homeostasis, cellular senescence, cell stemness, and proteostasis [37]. SIRT-1 also plays a key role in healthy aging and longevity [38], since it is involved in many physiological processes such as mitochondrial biogenesis, gene silencing, cell longevity, and metabolic regulation through deacetylation of histones and non-histone substrates [39]. Dietary restrictions may be involved in “longevity regulatory” pathways including IGF-1, mTOR, and sirtuins such as SIRT-1. Thus, although the molecular mechanisms remain not fully understood, the organism appears to respond to dietary restrictions minimizing cellular anabolic processes and enhancing stress resistance, resilience, and tissue repair. Given that this evidence is provided from animal model studies, future clinical trials are required to translate these promising findings to human physiology.

### 2.2. Cancer and Dietary Restrictions

Several animal studies have shown that CR and fasting interventions reduce the occurrence of tumors during normal aging [31,40,41]. Cell proliferation is tightly regulated by the availability of nutrients, particularly glucose and glutamine, used by cancer cells to produce adenosine triphosphate (ATP) and metabolites [42]. One possible explanation is that dietary restriction acts by altering the level of IGF-1, which works as growth factor for tumors [43]. Indeed, preclinical studies in breast, pancreatic, and colon cancer demonstrated that CR could modulate the IGF-1 signaling [44,45,46]. Prolonging fasting could also reduce IGF-1 levels in various cell populations, leading to signal transduction changes in long-term hematopoietic stem cells [47]. Additionally, during short-term fasting, in contrast to cancer cells, normal cells could be protected against chemotherapy-dependent damage by reducing circulating IGF-1 levels and through a downregulation of proto-oncogene signals [48,49]. Hence, short-term fasting may maximize the differential toxicity to normal and cancer cells during chemotherapy [48].

These promising preclinical results suggest that dietary restrictions may potentially attenuate the cytotoxic effects of cancer treatments in humans. In clinical practice, dietary guidelines during cancer treatment are based on the prevention of nutrient deficiencies to preserve muscle mass and overcome the side effects of treatments such as decreased appetite, nausea, taste changes, or bowel impairment. A CR-based strategy is potentially harmful since a chronic CR could increase the risk of weight loss, cachexia, and sarcopenia in cancer patients. However, short-term fasting may be feasible in selected patients [50]. To date, very few studies on fasting have been conducted in cancer patients. A recent study analyzed 10 patients diagnosed with a variety of malignancies (prostate, breast, ovary, and lung) who voluntarily fasted prior to (48–140 h) and/or following (5–56 h) chemotherapy [51] for an average of four chemotherapy cycles. None of them reported significant side effects caused by the fasting itself other than hunger and light-headedness [51]. A randomized pilot study [52] examined the effectiveness and safety of a short-term fasting on chemotherapy tolerance in early breast cancer patients and showed a reduction of hematological toxicities in the fasting arm compared to controls. Dorff et al. studied three cohorts of cancer patients who fasted for 24, 48, and 72 h before platinum-based chemotherapy, demonstrating that fasting for 72 h around chemotherapy administration may be safe and feasible for cancer patients [53]. The authors went further, demonstrating that IGF-1 levels could be a potential biomarker of chemotherapy toxicity [53]. However, randomized controlled trials with a higher sample size are required to assess the efficacy and safety of fasting during cancer treatment [54].

### 2.3. Neurodegenerative Disorders and Dietary Restrictions

CR and fasting influence brain metabolism and functions, reducing the amount of glucose necessary to maintain neuronal activities. After few hours of fasting (up to 20 h), a metabolic switch occurs, characterized by liver production of ketones, representing the main fuel for neurons. In hippocampal and cortical neurons, the primary blood ketone, beta-hydroxybutyrate, plays a key signaling role by inducing the transcription of brain-derived neurotrophic factor (BDNF) via its inhibition of histone deacetylases [55]. BDNF is a pivotal regulator of neuron function, maintaining synaptic structure, stimulating the production and survival of new hippocampal neurons, and enhancing neuron resistance to disease [56]. In prokaryotes and laboratory animals, a daily 20–40% CR was shown to increase BDNF levels, thereby attenuating neurochemical and behavioral deficits and protecting against neurodegenerative disorders [57]. However, in clinical practice, the compliance and long-term tolerability of CR are low in neurodegenerative patients. Short-fasting periods such as IF or periodic fasting could be feasible, especially in Alzheimer’s and Parkinson’s patients [58,59]. IF could increase neuronal stress resistance through multiple mechanisms, including bolstering mitochondrial function and stimulating autophagy, BDNF production, antioxidant defenses, and DNA repair [59]. Human-controlled trials of short-fasting interventions are needed to assess the long-term effects of the intervention on the progression of the disease.

Multiple sclerosis (MS) is the most common chronic inflammatory autoimmune disease of the central nervous system in young adults [60]. MS is characterized by an accumulation of demyelinating lesions and a neuronal degeneration in the central nervous system [60]. In a murine MS model (experimentally-induced autoimmune encephalomyelitis, EAE), periodic 3-day cycles of a FMD are effective in ameliorating demyelination and symptoms [61]. Indeed, Choi et al. showed that the FMD reduced clinical severity in all EAE mice, and completely reversed symptoms in 20% of the animals. Moreover, the FMD could promote oligodendrocyte precursor cell regeneration and remyelination in axons, supporting its effects on both suppression of autoimmunity and remyelination [61]. In humans, a recent randomized controlled trial studied 111 MS patients following a 7-day fast every 6 months, with 14 h daily intermittent fasting in between the fasts, for a period of 18 months [62]. This study confirmed preclinical results suggesting FMD is safe, feasible, and potentially effective in the treatment of relapsing, remitting MS patients [62].

### 2.4. Obesity, Type 2 Diabetes, Cardiovascular Diseases, and Dietary Restrictions

Obesity is defined as an abnormal or excessive fat accumulation that presents a risk to health [3]. To date, in obese animal and human models [63,64,65,66,67,68], CR remains the cornerstone intervention to decrease the loss of fat mass. Moreover, various short-term studies showed that IF could also be as effective as daily CR in producing weight loss [69]. Interestingly, in mice, IF could be associated with white adipose browning [14].

Obesity and inflammation open the door to insulin resistance and type 2 diabetes [70]. In rats, FMDs have been also shown to improve insulin sensitivity and ameliorate diabetic retinopathy [71]. In humans, two studies showed that CR or IF could improve insulin sensitivity in patients with prediabetes or type 2 diabetes [72,73].

In overweight/obese humans, numerous randomized controlled trials have shown that CR and fasting interventions improve multiple indicators of cardiovascular health, including blood pressure [74,75,76,77], levels of high-density (HDL) [74,75,76,78], low-density lipoprotein (LDL) [74,75,76,78,79], cholesterol [74,75,76,77,78,79,80], and triglycerides [74,75,76,77,78,79,80]. In addition, in rodents, CR has been shown to delay the development of atherosclerotic lesions [81]. Specifically, CR could induce SIRT-1 activation involved in the cardiac function through angiogenic activity [82] and the regulation of contractile function in cardiac muscle [83].

Therefore, many preclinical and clinical studies suggest that CR and fasting diets provide various health benefits related to NCDs such as cancer, neurological disorders, obesity, diabetes, and cardiovascular diseases. Although the specific biological mechanisms are not fully understood, gut microbiota variations can be involved in the beneficial effects of CR on NCDs.

## 3. Dietary Restrictions and Gut Microbiota Variations

### 3.1. Bidirectional Interplay between Aging and Gut Microbiota Variations

The gut microbiota is composed of microorganisms, including yeast, parasites, viruses, and different bacteria species taxonomically classified by genus, family, order, and phyla. Each human’s gut microbiota is unique and shaped in early life [84,85]. The microbial colonization of the gut starts from the time of birth and reaches maturation within the first two years. The composition of the core native gut microbiota mainly depends on infant transitions such as the type of delivery, methods of milk feeding, weaning period, and external factors such as antibiotic use. After birth, gut microbiota is dominated by Lactobacillus and *Prevotella* spp. for vaginally-delivered infants while Staphyloccocus, Corynebacterium, and *Propionibacterium* spp. are predominant in infants delivered by caesarean section [86]. During milk feeding, the infant’s gut microbiota is mainly colonized by *Bifidobacterium* spp. and, after weaning by Bacteroides, Prevotella, Ruminococcus, Clostridium, and *Veillonella* spp. [87]. Gut microbiota remains relatively stable in adulthood but differs between individuals due to exercise frequency, lifestyle, and cultural and dietary habits [88]. Richness and diversity of gut microbiota shaped in early life characterize a healthy adult gut microbiota composition. Several studies [89,90] on aging and gut microbiota have revealed that the composition of microbiota in the elderly is significantly different from that of younger adults. Specifically, lower abundance of phylum Firmicutes and an overall lower microbial diversity were detected in older subjects compared with young subjects [89]. Indeed, immunosenescence, defined as a decline in the functionality of the immune system, is correlated with unfavorable changes in the composition and structure of the gut microbiota in older people [91,92]. Furthermore, these compositional variations are associated with incidences of several chronic diseases, but it remains unclear whether gut microbiota alterations are the cause or consequence of aging diseases [85]. Alterations of gut microbiota could be attributed to several reasons such as modifications of lifestyle and dietary schedule, lesser mobility, immune system deficiency, altered gut morphology and physiology, recurrent infections, hospitalizations, and use of medications [93]. In this context, a growing number of animal and human studies reported the effect of different dietary restrictions on gut microbiota composition.

### 3.2. Caloric Restriction (CR) and Gut Microbiota Variations

CR could impact all the main phyla of gut bacteria such as Firmicutes, Bacteroidetes, Proteobacteria, Verrucomicrobia, and Actinobacteria. In the Firmicutes phylum, several studies assessed the impact of 25–40% CR based on a normal ad libitum diet and showed an increase of Lactobacillaceae [16,94], such as *Lactobacillus* spp. [17,18,19,20,21,22], Erysipelotrichaceae [16,94], and Ruminococcaceae [94] in CR rodents compared with normal-diet rodents. Conversely, a decrease of *Lactococcus* spp., [22], Lachnospiraceae [22,95] and Clostridiales [94,95] was found in CR rodents compared with controls.

CR could also impact the composition of Bacteroidetes, increasing the abundance of Bacteroidaceae [16,17,20,21,94], Proteobacteria [17,21,22,96], and Verrucomicrobia [16,96] with an increase of *Akkermansia muciniphila* counts [16]. In the Actinobacteria phylum, in several studies [17,20,21,22], counts of some *Bifidobacterium* spp. increased in CR animals compared with normal diet-fed animals.

Interestingly, a recent study by Zhang et al. [97] analyzed the effect of CR timing on gut microbial variations in mice. Compared to light-fed CR, dark-fed CR brought potentially beneficial structural shifts in the gut microbiota with an increase of counts of *Lactobacillus murinus* and Roseburia whereas light-fed CR induce an increase of abundance of Helicobacter and Alistipes [97].

In human studies, gut microbiota variations were also observed in CR group compared with control group. In obese women following a 4-week very-low calorie diet (VLCD, 800 kcal/day), CR was associated with an increased occurrence of *Ruminococcus* spp. *Anaerostipes hadrus,* and *Bifidobacterium* spp. [23] and a decrease of Proteobacteria. Furthermore, a 10-week energy-restricted diet in overweight adolescents increased counts of *Bacteroides fragilis* and decreased counts of *Blautia coccoides* and *Bifidobacterium longum* [98]. Similarly, after a 10-week of VLCD, obese adolescents reported an increased number of the *Bacteroides* spp. [99]. Another study in obese adolescents following CR for one year confirmed a growth of *Bacteroides* spp. *Roseburia* spp. *Faecalibacterium* spp. and *Clostridium XIVa* [100]. Conversely, CR induced Coriobacteriaceae, *Streptococcus* spp., Clostridiales, *Eubacterium* spp., *Coprococcus* spp., decreased in abundance [100].

In summary, CR induces alterations in the relative abundances of microbial families. However, these changes varied between studies. These discrepancies could be explained by the high heterogeneity in terms of inter-individual variations, study model, and different analysis techniques of microbiota composition.

### 3.3. Fasting and Gut Microbiota Variations

Several animal studies reported that fasting interventions could also shape gut microbiota. A recent obese murine model compared the effects of high-fiber diet, CR, IF, and TRF on gut microbiota composition [101]. Compared with the control group, an increase of Ruminococcus, Christensenellaceae, Clostridiales, *Coprococcus* spp. and *Lactococcus* spp. was shown in the TRF group, an increase of Bifidobacterium in the IF group, and an enrichment of Bacteroidetes in all fasting groups [101]. Inversely, Bilophila abundance decreased in all fasting groups as well as *Enterococcus* spp. and *Lactococcus* spp., in the IF group. Given that the strategies of TRF can differ according to different daily hours of fasting, a recent study investigated the effects of 12, 16, and 20 h daily fasting for 1 month on gut microbiota in mice [102]. The variations of gut microbiota induced by TRF were more significant in mice treated with daily 16h fasting with a decrease of Ruminococcaceae and Alistipes abundance and an increase of *A. muciniphila* counts compared with control [102].

Moreover, various studies in type 2 diabetic mice showed that IF regimen could stimulate the enrichment of species of the genera Lactobacillus [103,104], Oscillospira [103], and Ruminococcus [103] and the reduction of *A. muciniphila* [103], Bacteroides [103], Bifidobacterium [103], Enterococcus [104], and Streptococcus [104], compared with ad libitum feeding. Furthermore, compared with control mice, FMD-diabetic mice showed an increase of Parabacteroides and *Blautia* abundances and reduced counts of Prevotellaceae, Alistipes and Ruminococcaceae [15].

In a mouse model of MS, IF changed gut microbiome composition resulting in increased bacteria richness and enrichment of Lactobacillaceae, Bacterioidaceae and Prevotellaceae families and *Bifidobacterium pseudolongum* [105]. Futhermore, a recent murine model mimicking an IBD syndrome investigated 4-day of FMD reporting an expansion of Lactobacillaceae and Bifidobacteriaceae [12].

As regards human studies, a pilot study [106] examined fecal microbiota in overweight people undergoing a fasting program (with laxative treatment) for 1 week followed by a 6-week intervention with a probiotic formula. Compared to baseline stool samples, no significant changes of total bacteria, Bacteroidetes, Prevotella, *Clostridium cluster XIVa*, or *Clostridium cluster IV* abundance were found whereas an increase of *Faecalibacterium prausnitzii*, *A. muciniphila,* and *Bifidobacteria* spp. abundance was shown over the study period [106]. Moreover, Enterobacteria and Lactobacilli abundances increased during the first week and then declined by the end of the intervention. Recently, the well-known Ramadan fasting, corresponding to 17 h of fasting/day during 29-day period, was investigated [24]. A significantly-increased abundance of *A. muciniphila* and *Bacteroides fragilis* was found after the Islamic fasting period [24].

All these animal and human studies demonstrated a strong relationship between dietary restriction and gut microbiota composition with changes in the types and numbers of gut bacteria. These gut microbiota changes are not just associated with dietary restriction but could drive several health benefits linked to NCDs.

## 4. Gut Microbiota Changes as a Potential Driver of Health Benefits during Dietary Restriction

Several studies correlated gut microbiota compositional changes with host health, in particular gut barrier functions, metabolism, and brain functions, during dietary restrictions (Table 1).

### 4.1. Gut Microbiota and Gut Barrier Permeability during Dietary Restrictions

#### 4.1.1. Gut Barrier Permeability and Immune System

The intestinal barrier represents a functional unit responsible for two main tasks that are crucial for the survival of the individual—allowing nutrient absorption and defending the body from the penetration of unwanted, often dangerous, macromolecules. The gut mucosa is a multi-layered system consisting of an “anatomical” barrier and an inner “functional” immunological barrier [107]. Commensal gut microbiota, the mucus layer, and the intestinal epithelial monolayer constitute the anatomical barrier [108]. However, in recent decades, it has become evident that gut barrier is much more than a physical barrier. Indeed, intestinal epithelial cells are important sources of cytokines that regulate functions of various cell types in the intestinal mucosa such as regulatory T (Treg) cells [109]. The inner layer consists of a complex network of immune cells organized in a specialized and compartmentalized system known as gut-associated lymphoid tissue (GALT). GALT represents both isolated and aggregated lymphoid follicles, and is one of the largest lymphoid organs, containing up to 70% of the body’s total number of immunocytes. Moreover, it is involved in the response to pathogenic microorganisms and provides immune tolerance to commensal bacteria. The ability of GALT to interact with luminal antigens rests on specific mucosal immune cells (i.e., dendritic cells and M-cells), primarily localized to Peyer’s patches within the ileum that are intimately positioned at the mucosa–environment interface and internalize microorganisms and macromolecules [110]. These specialized immune cells have the ability to present antigens to naïve T-lymphocytes, which subsequently produce cytokines and activate mucosal immune responses, when needed. From the intracellular point of view, inflammasomes are a group of protein complexes that assemble upon recognition of a diverse set of noxious stimuli and are now considered the cornerstone of the intracellular surveillance system. Inflammasomes are able to sense both microbial and damage-associated molecular patterns (DAMPs) and initiate a potent innate, anti-microbial immune response [111]. In this context, dysregulation of gut permeability with bacterial translocation of luminal contents to the underlying mucosa impacting immune system is implicated in the pathogenesis of several diseases such as IBD, irritable bowel syndrome (IBS), and chronic liver disease [112].

#### 4.1.2. Gut Barrier Immunity, Gut Permeability, and Aging

Colonic biopsies from old baboons showed an up-regulation of microRNA and inflammatory cytokines interferon (IFN)-γ, interleukin (IL)-6, and IL-1β, compared with young animals [113]. The increased levels of these inflammatory mediators may have a direct impact on the aging of gut, as cytokines induce dysregulation of the tight junction barrier resulting in increased gut permeability [114]. Indeed, IL-1β was shown to cause an increase in intestinal epithelial tight junction permeability, via the activation of both canonical and non-canonical pathways in intestinal epithelial cells [115]. Interestingly, studies in Drosophila demonstrated that impairment of intestinal barrier function predicted age-onset mortality [116], suggesting that intestinal barrier dysfunction may be an important factor in the pathophysiology of aging. However, a recent human study [117] assessed the effects of aging on intestinal barrier function in humans in vivo and ex vivo, showing no significant differences between healthy young and elderly IBS patients for small intestinal, colonic, and whole gut permeability. All these findings need to be confirmed and thoroughly investigated to understand the clear mechanisms between gut barrier permeability and aging.

#### 4.1.3. Gut Microbiota and Gut Barrier Permeability during Dietary Restrictions

The Lactobacillus genus is well-known to promote intestinal homeostasis by stimulating host signaling pathways and the immune system [118]. A study [18] analyzed fecal samples of ad libitum fed young rats after 8 weeks of CR and showed a significant increase of the *Lactobacillus* spp. in the CR group. In particular, a mouse model study [19] assessed an enrichment of two strains of *L. murinus* induced by CR. This study went further, investigating the role of *L. murinus* (in an in vitro model and in vivo in a *Caenorhabditis elegans* model) and reported that *L. murinus* isolated from the feces of CR mice was one of the key members contributing to the protection of the gut barrier and the attenuation of chronic systemic inflammation. As previously described, in IBD mice, FMD could also increase Lactobacillaceae counts [12].

The Bifidobacteriaceae family was also shown to have protective effects on intestinal barrier function and to improve symptoms in colitis mice model [119]. CR and fasting diets could lead to an increase of Bifidobacteria abundance [21,23,98,101]. Specifically, in the IBD mice model [12], Bifidobacteriaceae was found to be enriched in the FMD group compared to the control. Rangan et al. also performed a fecal transplant from FMD-treated mice and showed positive changes in IBD-associated symptoms [12].

Moreover, *A. muciniphila* is a mucin-degrading bacterium of the phylum Verrucomicrobia and accounts for 3% of human gut microbiota [120]. Several studies in mice demonstrated that, compared to control, *A. muciniphila* increased in abundance during CR [16] and during TRF [102]. Such evidence has also been confirmed in humans during Islamic fasting [24]. In most human studies, a decrease of *A. muciniphila* was observed in IBD mucosa and fecal samples from ulcerative colitis patients [121,122] and in those with cirrhosis and hepatocellular carcinoma showing increased markers of intestinal inflammation [123], suggesting that it may have protective and anti-inflammatory effects. Its mucolytic property also has other beneficial effects, since it leads to the production of oligosaccharides, amino acids, propionate, acetate, and important vitamins and cofactors, which become useful for other microbial commensals [124,125]. Emerging studies on animal models have indicated its ability to modulate genes implicated in immune-response regulatory processes [126]. *A. muciniphila* can also release vesicles with an anti-inflammatory activity on intestinal cells and dampen the severity of colitis in mice [127]. Moreover, despite its mucolytic nature, *A. muciniphila* could stimulate mucin production and enhance anti-inflammatory Treg proliferation and improve gut barrier integrity [128]. Indeed, a recent report showed that *A. muciniphila* decline may represent a definitive biomarker of dysbiosis shared by patients with different gastrointestinal and extraintestinal autoinflammatory diseases and be the most relevant discriminating factor able to dissect the complex equilibrium between the health and disease status [129]. These data emerged from an unsupervised analysis focused only on microbiota composition, but then were further supported by a supervised approach including *A. muciniphila* levels from both patients and healthy controls. In this scenario, *A. muciniphila* decline was associated to a decrease of *Bifidobacterium* spp.. Remarkably, both these bacteria have been inversely correlated with several metabolic disorders, as well as inflammation and insulin resistance [130,131]. *A. muciniphila* reduction could be the basis of dysbiotic events that lead to the elevation of specific proinflammatory species able to overcome its protective effects. Beside this, the dysbiotic decline of *A. muciniphila* was associated with a decrease of important metabolic pathways independently of their clinical condition [129]. Subjects characterized by a low level of *A. muciniphila* presented a reduction of tryptophan metabolism pathway, thus probably with a reduction of indole and indole derivatives catabolism [132]. Indole plays a crucial role in the maintenance of mucosal homeostasis and barrier functions, and recent evidence have shown that *A. muciniphila* is directly involved in the improvement of gut-barrier functions [133]. Subjects with a decline of *A. muciniphila* also showed a reduction of xenobiotic metabolism, which has important pharmacokinetic properties [134]. The identification of the relationship between human gut microbiota composition and disease represents the basis for planning more rational microbiota-oriented therapeutic strategies. These observations provide a strong motivation for the selection of this strain and for the optimization of combined dietary approaches to efficiently modulate its relative abundance, with the final aim of improving nutritional and clinical practice.

Overall, specific dietary restriction could impact *Lactobacillus* spp., *Bifidobacterium* spp., and *A. muciniphila* richness and diversity that could contribute to preserving or restoring gut barrier permeability and enhancing gut anti-inflammatory responses in IBD.

### 4.2. Gut Microbiota and Host Metabolism during Dietary Restrictions

Increased abundances of Firmicutes at the expense of Bacteroidetes were described in obese subjects compared with lean subjects [135]. When these subjects were submitted to a CR for one year, they underwent to an increase of their Bacteroidetes abundance and the normalization of their Firmicutes/Bacteroidetes ratio, in parallel with weight loss [135].

A recent study by Wang et al. [21] employed an antibiotic-induced microbiota-depleted mouse model to investigate the role of gut microbiota in anti-obesity effects of CR. The authors confirmed that CR altered the composition of gut microbiota with a significant increase in *Lactobacillus* spp. and *Bifidobacterium* spp. abundance and decrease in Firmicutes (Lachnospiraceae, Oscillibacter, Roseburia), Actinobacteria (Gordonibacter) and Proteobacteria (Helicobacter) abundances [21]. Of note, they showed that the depletion of gut microbiota rendered mice resistant to CR-induced loss of body weight, and subject to increase in fat mass, reduction in lean mass, and increase in fasting blood glucose and cholesterol levels [21]. Then, they performed fecal microbiota transplantation (FMT) on mice fed with a high-fat diet. Mice with transferred microbiota from CR mice resisted to high fat diet-induced obesity and exhibited metabolic improvements such as glucose tolerance, reduced leptin levels, and loss of body weight with a decrease in fat mass and increase in lean mass [21].

Several studies found an enrichment of *Lactobacillus* spp. induced by different dietary restrictions in mice [17,20] and humans [98]. An interesting study [20] compared the effects of the administration of the feruloyl esterase-producing strain *Lactobacillus fermentum* CRL1446 to a CR diet, compared to a control diet and a CR diet. Compared with the control, CR-diet plus *Lactobacillus fermentum* mice and CR-diet mice had an improvement of oxidative and metabolic parameters such as triglycerides, total cholesterol, and glucose levels. Moreover, CR with *Lactobacillus fermentum* CRL1446 administration increased abundance of Bifidobacterium and Lactobacillus genus, which was correlated with improved metabolic parameters [20].

As previously described, a decrease in Firmicutes such as Lachnospiraceae was shown in various CR and fasting studies [21,23,98,101]. A study [136] identified a bacterium (AJ110941) belonging to Lachnospiraceae in the feces of hyperglycemic obese mice. The colonization of germ-free obese mice by Lachnospiraceae induced significant increase in fasting blood glucose levels as well as liver adipose tissue weight, and reduced plasma insulin levels [137]. These results indicated that Lachnospiraceae could influence the development of obesity and diabetes [137].

To investigate the role of gut microbiota during CR in glucose tolerance and insulin sensitivity, Fabbiano et al. [16] performed an FMT from mice on 6-week CR dietary regimens to germ-free mice. The CR-FMT was sufficient to improve glucose tolerance after oral glucose gavage and to increase insulin sensitivity [16]. Interestingly, CR-microbiota-transplanted mice showed less weight gain compared to the ad libitum transplanted controls despite no changes in food intake and caloric uptake. Moreover, *A. muciniphila* abundance increased in CR mice [16], as previously described by another study [106]. Of note, studies showed that *A. muciniphila* abundance is higher in lean compared with obese and diabetics individuals [114,138]. Furthermore, weight loss could increase the abundance of this bacterium [106] and reduce insulin sensitivity [139,140].

In another study [94], Duska et al. studied mice transplanted with fecal microbiota from CR mice and found decreased concentrations of all short chain fatty acids (SCFAs). Furthermore, a rat model study [141] showed that a switch from ad libitum low fat diet to CR in young rats is able to induce a significant change in the expression of the microbial enzymes responsible for SCFA biosynthesis, particularly with a CR limitation of butyrogenesis [141]. It can be argued that decreased butyrate levels may play a signaling role in CR by preventing stimulation of adipocyte proliferation and attenuating signaling of satiety [142].

On the other hand, Li et al. demonstrated that IF resulted in a shift in the gut microbiota composition leading to the elevation of the fermentation products acetate and lactate, promoting white adipose browning [14]. Browning of white adipose cells activates changes in energy homeostasis, countering the effects of elevated energy intake and resulting in metabolic improvements. In the study of Li et al., microbiota-depleted mice were resistant to IF-induced browning of white adipose tissue, while FMT from IF-treated mice to microbiota-depleted mice activated browning of white adipose cells [14]. These findings provided a potential gut microbiota-driven mechanism for activating adipose tissue browning and treating metabolic diseases [14]. A recent study confirmed these findings and showed that microbiota remodeling is an important contributor of brown fat induction during CR and a key factor in metabolic improvements caused by CR, including improved insulin sensitivity, glucose tolerance, and lowered fat gain [16].

Thus, CR or fasting interventions induce compositional gut microbiota changes leading to improvements in loss of body weight and fat mass, lipid metabolism, glucose tolerance, and insulin sensitivity. Furthermore, through these metabolic benefits, microbiota changes induced by dietary restriction could slow the onset of some NCDs such as obesity, diabetes, and cardiovascular diseases.

### 4.3. Gut Microbiota and Brain Functions during Dietary Restrictions

The brain and the gut are connected with bidirectional interactions between the central nervous system, the enteric nervous system, and the gastrointestinal tract (the so-called “gut–brain axis”). Microbiota homeostasis is essential for modulating cognitive functions via the regulation of the permeability of the blood–brain barrier, brain energy homeostasis, brain development, and finally behavior [143].

It is known that chronic stress significantly alters intestinal microbiota composition, primarily depleting Lactobacilli [144]. A recent study by Liu et al. [104] investigated the role of gut microbiota changes induced by IF in mediating inherent diabetic cognitive deficits. First, they found that the 28-day IF regimen improved gut barrier integrity and decreased the plasma lipopolysaccharides (LPS) level, which could partly explain how IF reduces neuro-inflammatory responses. IF also impacts microbiome diversity in diabetic mice, with alteration of gut microbiota abundance, particularly enhancing the abundance of Lactobacillus. It has been shown that the administration of *Lactobacillus reuteri* to stressed mice could improve metabolic homeostasis and correct stress-induced despair behaviors [144]. Secondly, they assessed whether the beneficial effects of IF could be affected after removing the gut microbiota in diabetic mice and found that antibiotic treatment partly abolishes the neuroprotective effects of IF [104].

As previously described, IF could influence microbiota composition variations in MS animal models with an increase of the abundance of the Bacteroidaceae, Lactobacillaceae, and Prevotellaceae microbial families in the IF group compared to the ad libitum group [105]. The IF-induced enrichment in Lactobacilli, which is commonly used in probiotics could lead to positive effects including reduction of inflammatory immune responses [105]. The enrichment in Prevotellaceae induced by IF may be beneficial since Prevotellaceae could enhance the production of SCFAs, including butyrate [145] reported to inhibit autoimmune encephalomyelitis by expanding gut Tregs [105]. Although IF can influence gut microbiota with consequent potential benefits in MS, these results need to be confirmed in a larger clinical study to test IF and microbiome manipulation as a potential treatment in multiple sclerosis.

Alzheimer’s disease (AD) is another model for the study of the gut–brain axis. The main hallmark of AD is the plaque deposits of the β-amyloid peptide (Aβ). Indeed, familial early-onset forms of AD are associated with mutations in the β-amyloid precursor protein (APP). In AD patients, levels of Bacteroides are elevated and correlated with blood levels of Aβ [146]. Recently, Cox et al. studied the microbiota changes of transgenic mice with early-onset AD due to the insertion of a mutant variant of the human APP [13]. This model undergoes cerebral amyloid accumulation, synaptic loss, and cognitive impairment. Under CR (30% reduction in carbohydrates only), transgenic mice could substantially change their microbiota normalizing the increase of *Bacteroides* spp. [13]. Moreover, the authors showed that *Bacteroides fragilis* could increase Aβ plaque deposition in the brain, a mechanism whereby gut microbiota impacts AD pathogenesis [13]. Hence, CR may alter gut microbiota and prevent the expansion of bacteria such as *Bacteroides* spp. that contribute to age-related cognitive decline such as AD. However, the same study went further and revealed that CR could lead to higher mortality in aged mice in contrast to lengthening lifespan in younger mice [13].

**Figure 1:**
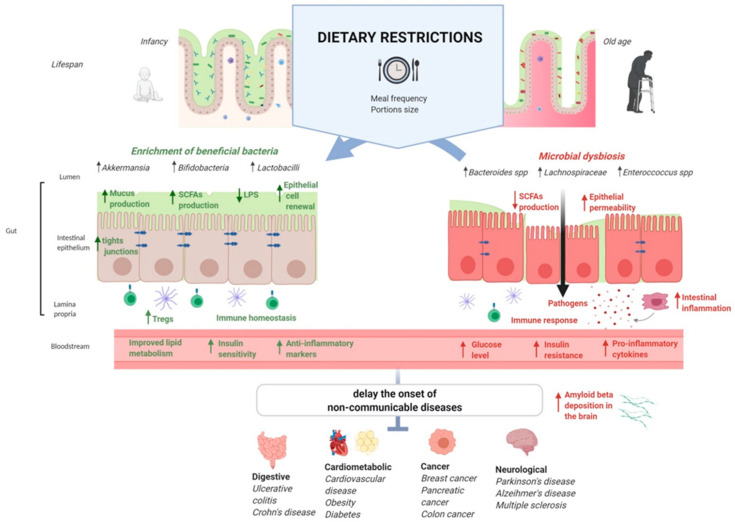
The possible role of gut microbiota in the interplay between dietary restrictions, gut barrier functions, health benefits, and non-communicable diseases. Abbreviations: LPS, lipopolysaccharide; SCFAs, short-chain fatty acids.

These findings show that dietary restriction could represent potential therapeutic strategies against chronic diseases through the modulation of microbiota (Figure 1). More specifically, FMT studies [12,16,21,94] have provided evidence for a causal role of gut bacteria in the modulation of metabolic, cardiovascular, and immune phenotypes involved in the pathogenesis of various NCDs.

## 5. Conclusions

CR and fasting diets conducted under clinical supervision are safe, relevant, and inexpensive additional clinical interventions to treat or prevent these NCDs. Dietary restriction could slow down compositional age-microbiota changes with an enrichment of beneficial bacteria, which may positively influence host metabolism, immunity, gut barrier, and brain functions, although the current available scientific evidence mainly relies on animal studies. Through the modulation of microbiota, these induced health benefits may delay the onset of NCDs and prolong the health span and lifespan. In particular, the impact of dietary restriction on the gut microbiota has a key role in diabetic and obese individuals, as gut dysbiosis may already induce an increased risk of systemic inflammation. However, the molecular cascades between dietary restrictions, gut microbiota, and host health remain to be elucidated and further human studies are required to better understand the microbiota-driven mechanisms and implications on NCDs. These new insights could lead to the development of novel strategies such as dietary restriction approaches to modulate the microbiota and treat or prevent these diseases. To date, such scientific evidence confirms the healthy role of fasting for the body, as most religions have suggested for many centuries for the spirit.

## Figures and Tables

**Table 1:** Overview of studies investigating the possible correlations between gut microbiota variations, gut barrier permeability, and health benefits induced by dietary restrictions.

Study	Dietary Restriction Regimen	Study Model	Gut Microbiota Variations Induced by Dietary Restrictions					Effects on Gut Barrier Functions	Potential Health Benefits
			Firmicutes	Bacteroidetes	Proteobacteria	Verrucomicrobia	Actinobacteria		
			**Caloric restriction studies**						
Zhang, 2013 [22]	30% CR based on a low-fat AL diet	Mid-life mice	↓ Lactococcus ↑Lactobacillus	↓ Prevotellaceae	↑ Helicobacter	/	↑Bifidobacterium	/	↓ Serum levels of LPS-binding protein
	30% CR based on a low-fat AL diet	Late-life mice	↓ Lactococcus ↓ Lachnospiraceae ↑ Lactobacillus	↓ Bacteroides ↓ Parabacteroides	↓ Bilophila	/	↑ Bifidobacterium		
	30% CR based on a high-fat AL diet	Mid-life mice	↓ Peptostreptococcaceae	/	↓ Bilophila	/	/		
Russo, 2016 [20]	ALF group CR group (25% less than the daily ration) CR diet plus *L. fermentum* group (CR-Lf group) for 45 days	Mice	↑ Lactobacillus (CR group)	↑ Bacteroidetes	/	/	↑ Actinobacteria in CR and CR-Lf groupscompared to ALF group	/	↓ Triglyceride levels ↓ Total cholesterol levels ↓ Glucose levels ↑ Plasmatic glutathione reductase activity
Bartley, 2017 [96]	30% CR based on a normal AL diet	Mice with flu infection	/	/	↑ Proteobacteria	↑ Verrucomicrobia	/	/	↓ Flu-induced systemic inflammation
Duszka, 2018 [94]	25% CR based on a normal AL diet for 14 days	Mice	↓ Clostridiales ↑ Lactobacillaceae ↑ Lachnospiraceae ↑ Ruminococcaceae ↑ Erysipelotrichaceae	↑ Bacteroidaceae ↑Porphyromonadaceae ↑ Prevotellaceae	/	/	/	Downregulation of the metabolic and immune/inflammatory pathways Inhibition of the mTOR pathway	/
Fabbiano, 2018 [16]	40% CR based on a standard diet for 30 days	Mice	↑ Lactobacillaceae ↑ Erysipelotrichaceae	↑ Bacteroidaceae	/	↑ *Akkermansia muciniphila*	/	/	↑ Glucose tolerance ↑ Insulin sensitivity ↓ Weight gain ↓ Fat volume/mass ↓ Number of adipocytes ↑ Browning of the white fat depots
Fraumene, 2018 [18]	30% CR based on a normal AL diet	Rats	↑ Lactobacillus	/	/	/	/	/	↓ Total cholesterol levels ↓ Triglyceride levels
Pan, 2018 [19]	30% CR based on a normal AL diet	Mice	↑ Lactobacillus	/	/	/	/	↓ Gut barrier permeability	↓ Aging-associated inflammation
Wang, 2018 [21]	70% of normal chow based on the food intake of control group	Mice	↑ Lactobacillus ↓ Firmicutes	↑ Bacteroidetes	↓ Helicobacter	↓ Verrucomicrobia	↑Bifidobacterium ↑ Actinobacteria	/	↓ Weight gain ↓ Body fat mass ↑ Glucose tolerance ↓ Fasting blood glucose ↓ Blood leptin level
Fabersani, 2019 [17]	25% CR based on a standard diet 25% CR supplemented with *L. fermentum, L. casei,* or *L. lactis* for 45 days	Male mice	↑ Lactobacillus (CR + *L. casei*)	↑ Bacteroidetes	↓Proteobacteria	/	↑ Actinobacteria ↑ Bifidobacterium in CR + *L. fermentum* group	/	↓ Blood glucose levels ↓ Total cholesterol levels ↓ Triglyceride levels ↓ Leptin levels
Zeng, 2019 [95]	30% CR based on a standard diet Three groups: ALF young mice ALF old mice old mice fed with CR diet for 2 months	Young and old female mice	↓ Firmicutes ↓ Lachnospiraceae ↓ Clostridia ↓ Clostridiales	/	/	/	/	/	↓ Fat accumulation ↓ Inflammation ↓ Reduced body weight ↓ Abdominal fat
Zhang, 2019 [97]	4-week 30% CR based on a normal AL diet CRL group was fed at the beginning of the light phase CRD was fed at the beginning of the dark phase	Mice	↑ *Lactobacillus murinus* and Roseburia in CRD than CRL mice. ↑ *L. reuteri* and L. *gasseri* in CRL than CRD mice.	↑ Alistipes in CRL than CRD mice.	↑ *Helicobacter* spp. in CRL than CRD mice.	/	/	Improved intestinal barrier function	↓ Fat accumulation ↓ Fasting glucose level ↓ Inflammatory markers
Ott, 2017 [23]	4-week VLCD (800 kcal/ day)	Obese women	↑ *Anaerostipes hadrus* ↓ *Agathobacter rectalis* ↑ Ruminococcus	/	↓Proteobacteria	/	↑ *Bifidobacterium* spp.	↓ Gut barrier permeability	↓ High-sensitivity C-reactive protein ↓ LPS binding protein
Ruiz, 2017 [100]	30% CR based on usual diet for one year	Obese adolescents	↑ Roseburia ↑ Faecalibacterium ↑ *Clostridium XIVa* ↓ Clostridiales ↓ Streptococcus ↓ Eubacterium ↓ Coprococcus	↑ *Bacteroides* spp.	/	/	↓ Corobacterineae	/	↓ Plasma LDL levels ↓ Plasma insulin levels
Santa cruz 2009 [98]	10-40% CR diet and regular physical activity over 10 weeks	Overweight Adolescents	↑ Lactobacillus ↓ *Clostridium coccoides*	↑ *Bacteroides fragilis*	/	/	↓ *Bifidobacterium longum* ↓ *Bifidobacterium adolescentis*	/	↑ Body weight loss
Simoes, 2014 [99]	5-, 8- and 12- months of VLCD (800 kcal/day)	Obese adults	/	↑ *Bacteroides* spp.	/	/	↓ *Bifidobacterium*	/	
		**Fasting Diet Studies**							
Beli, 2018 [103]	7-month IF regimen	Diabetic mice	↑ Lactobacillus ↓ Oscillospira	↓ Bacteroides		↓ Akkermansia		↓ Gut barrier permeability	Protective effect of the retina ↑ Glucose tolerance
Cignarella, 2018 [105]	Every-other-day fasting regimen	Multiple sclerosis mice	↑ Lactobacillaceae	↑ Bacteroidaceae ↑ Prevotellaceae	/	/	↑ *Bifidobacterium pseudolongum*	↓ IL17 ↑ Treg cells Enhanced antioxidative pathways	↓ Leptin levels
Li, 2017 [14]	Every-other-day fasting regimen	Mice	↑ Firmicutes	/	/	/	/	↑Fermentation products acetate and lactate ↑ Metabolic homeostasis	Selective upregulation of beige cells
Li, 2020 [102]	Daily fasting: 12, 16, or 20 h fasting per day for 1 month	Mice	↓ Ruminococcaceae (16h fasting level)	↓ Alistipes (16h fasting level)	/	↑ Akkermansia (16h fasting level)	/	Alleviated intestinal inflammation	Metabolic improvements ↓ liver triglycerides
Liu, 2020 [104]	28-day IF regimen	Diabetic mice	↑ Lactobacillus ↓ Enterococcus ↓ Streptococcus ↓ unknown Enterococcaceae	↑ Odoribacter	/	/	/	↑ Gut barrier integrity	↓ Plasma LPS levels Neuroprotective effects
Merwe, 2020 [101]	HF–TRF (6 h) HF–IF HF-CR (80% total CR)	Mice	HF–TRF group: ↑ Ruminococcus ↑Christensenellaceae ↑ Clostridiales ↑ Coprococcus ↑ Lactococcus HF–IF group: ↓Enterococcus ↓ Lactococcus	↑ Bacteroidetes (HF–CR 16%, HF–TRF 20%, and HF–IF 14%)	HF–TRF group: ↑ Desulfovibrio ↓ Bilophila HF–IF group: ↓ Bilophila	↑ Verrucomicrobia	HF–IF group: ↑ Bifidobacterium	/	↓ Adiposity improved body composition ↑ Insulin sensitivity
Wei, 2018 [15]	Every-other-day fasting regimen For 8 weeks 7-day FMD alternating with 7 days of free eating	Mice with type 2 diabetes	↑ Blautia ↓ Ruminococcaceae ↓ Lachnospiraceae	↑ Parabacteroides ↓ Prevotellaceae ↓ Alistipes	/	/	/	/	↓ Fasting blood glucose levels ↑ Insulin sensitivity ↓ Obesity ↑ Glucose tolerance
Rangan, 2019 [12]	4-day FMD cycles	IBD mice	↑ Lactobacillaceae	/	/	/	↑ Bifidobacteriaceae	Inflammatory markers of intestinal inflammation Reversal of intestinal shortening ↑ *S*tems cells and regenerative effects	Partial reversal of intestinal inflammation
Ozkul, 2019 [24]	17 h of fasting/day during a 29-day period (fasting Ramadan)	Humans	/	↑ *Bacteroides fragilis*	/	↑ *Akkermansia muciniphila*	/		↓ Fasting blood glucose levels ↓ Total cholesterol levels
Remely, 2015 [106]	1-week fasting program followed by a probiotic administration	Humans	↑ *F. prausnitzii*	/	/	↑ *Akkermansia muciniphila*	↑ Bifidobacterium		Facilitated the adherence of probiotic-administered strains Improved gastrointestinal symptoms

Abbreviations: ↓ decrease of abundance; ↑ increase of abundance; AL, ad libitum; ALF, ad libitum fed; CR, caloric restricted; CRD, dark-fed caloric restriction; CRF, light-fed caloric restriction; FMD, fasting-mimicking diet; HF, high fat; IBD, intestinal bowel disease; IF, intermittent fasting; IL, interleukin; LDL, low-density lipoprotein; LPS, lipopolysaccharide; mTOR, mammalian target of rapamycin; Treg, regulatory T cell; TRF, time-restricted feeding; VLCD, very low calorie diet.
